# Perspectives on hand hygiene in Belizean healthcare facilities during the COVID-19 pandemic: a qualitative evaluation with healthcare workers

**DOI:** 10.2166/washdev.2024.317

**Published:** 2024-10

**Authors:** Kelsey McDavid, Christina Craig, Anh N. Ly, Nicholas Bivens, Francis Morey, Russell Manzanero, Gerhaldine Morazan, Ella Hawes, Alexandra Medley, Kristy Murray, Matthew Lozier

**Affiliations:** a Division of Foodborne, Waterborne, and Environmental Diseases, Centers for Disease Control and Prevention, 1600 Clifton Rd, Atlanta, GA 30333, USA; b Baylor College of Medicine and Texas Children’s Hospital, 1102 Bates Avenue, Suite 340, Houston, TX 70030, USA; c Belize Ministry of Health and Wellness, East Block Building, National Assembly, Bliss Parade, Belmopan, Belize

**Keywords:** focus group discussions, hand hygiene, healthcare, qualitative evaluation

## Abstract

The World Health Organization recommends healthcare workers (HCWs) practice hand hygiene (HH) while providing care. Making alcohol-based hand rub (ABHR) available at points of care is recommended during times of high patient volume, such as the COVID-19 pandemic. In low- and middle-income countries, such as Belize, there may be limited access to HH materials within healthcare facilities (HCF). This paper examines the motivators and barriers to HH among HCWs in the 11 largest public healthcare facilities in Belize and HCWs’ experiences with an intervention. In 2021, focus group discussions (FGDs) gathered HCWs’ HH perceptions and preferences. An intervention was then implemented to increase ABHR access and HH training for HCWs. Post-intervention endpoint FGDs in 2022 documented HCWs’ experiences with interventions. Baseline FGDs revealed that self-protection and protection of one’s household members from illness were key motivators for HCWs’ HH practice. Insufficient time, inadequate access to HH supplies, and gaps in education were barriers to practicing HH. At endpoint, participants appreciated increased access to ABHR and its convenience but did not like ABHR’s effect on hands. Experiences with the training were mixed. To improve HCWs’ HH practices, HH interventions should be tailored to HCWs’ context and learning preferences.

## Graphical Abstract

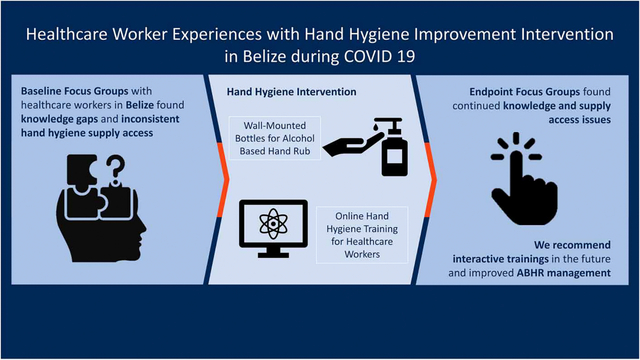

## INTRODUCTION

Healthcare-associated infections (HAIs) are a known risk for hospitalized patients, healthcare workers (HCWs), and visitors (Bardossy et al. 2016). In low- and middle-income countries (LMICs), HAI rates in inpatient settings can be as high as 25% (Allegranzi et al. 2011); there is a dearth of published data on HAIs for outpatient facilities. Frequent and proper hand hygiene (HH) has been identified as an important method for infection control in healthcare facilities (WHO 2009). It is a primary measure that the World Health Organization (WHO) recommends for preventing the spread of SARS-CoV-2 (WHO 2020). Without proper HH, HAIs can be transmitted between patients and HCWs.

The WHO recommends HCWs practice HH with alcohol-based hand rub (ABHR) or handwashing with soap and water (WHO 2009). Unfortunately, in LMICs, access to HH materials at points of care within healthcare facilities may be limited. A 2018 study assessing HCF environmental conditions in 78 LMICs found that only 50% of healthcare facilities had piped water, 61% had soap for handwashing, and 30% had ABHR (Cronk & Bartram 2018). While studies on HH and HAIs have been conducted in other LMICs, there are few published studies on the state of HH in public healthcare facilities in Belize (Bardossy et al. 2016). An assessment conducted in 2021 reported that four of the 11 largest public healthcare facilities in Belize did not have HH materials at 75% or more of points of care (Berendes et al. 2022). These findings suggest that HH access could be improved in Belizean healthcare facilities.

Previous studies show that internal and external factors beyond access to HH stations and materials can impact HH behaviors among HCWs (McLaughlin & Walsh 2012). The COM-B (Capability, Opportunity, Motivation, and Behavior) model divides the drivers of health behaviors, such as HH, into domains of capability (knowledge and skill), opportunity (physical and social), and motivation (reflective and automatic) (Michie et al. 2011). This model was used to explore HH behavior among HCF staff in the Dominican Republic during the COVID-19 pandemic, where motivation and opportunity were observed to be the greatest barriers to consistent HH practice (Craig et al. 2024).

The WHO developed the Multimodal Hand Hygiene Improvement Strategy, which recommends the implementation of multimodal interventions to improve HH practices, specifically focused on five domains: system change, training and education, monitoring and feedback of HH performance, workplace reminders, and creation of a HH safety culture (WHO 2009). The multimodal approach incorporates multiple drivers for HH practice to promote sustained behavior change.

A systematic review of qualitative studies in high-income countries found that motivational factors and perceptions of the work environment were primary elements influencing HCWs’ HH behavior, whereas a study conducted in eight LMICs found that access or lack of access to handwashing stations (physical opportunity) was the greatest self-reported factor driving HH behavior (Borg et al. 2009; Smiddy et al. 2015). There is no existing literature that explores the drivers and barriers to HH among HCWs in Belize and how these impact practices.

By integrating the theoretical framework of the COM-B model and elements of the WHO HH improvement strategy, this study uses qualitative methods to explore drivers of and barriers to HH among HCWs before and after implementing a two-part HH intervention. By understanding these drivers and barriers, healthcare facilities with similar resource constraints can better design interventions to address this challenge.

## METHODS

### Setting and study design

Belize is a small, upper-middle-income Central American and Caribbean country with a population of approximately 400,000 (World Bank 2023). The healthcare system in Belize is two-tiered, comprising a small private and large public system. The public healthcare system, led by the Ministry of Health and Wellness (MOHW), is made up of hospitals, polyclinics, and many smaller health centers and posts. All 11 public healthcare facilities (4 hospitals and 7 polyclinics) that participated in an acute febrile illness surveillance system implemented by the Baylor College of Medicine (BCM) were selected for this study (Shih et al. 2022). The healthcare facility settings ranged from outpatient clinics and small community hospitals (16 beds) to the national referral hospital (115 beds), and each of the six government districts of Belize was represented by at least one facility.

This study was part of a larger evaluation of HH in healthcare facilities. The HH evaluation included comprehensive quantitative baseline assessments of adherence to and resources and infrastructure for HH at the 11 healthcare facilities conducted in July 2021. Aggregate findings from the baseline quantitative assessments and focus group discussions (FGDs) were shared with the facilities and informed intervention design. A two-part intervention was developed and implemented, and an endpoint assessment was conducted after the intervention in June 2022. FGDs were conducted at each of the 11 participating facilities at baseline and endpoint.

### Participants and sampling

FGD participants were selected using a convenience sampling approach; the moderator aimed to include staff of different roles based on their availability on the day of the FGD. Each group consisted of 6–8 healthcare facility staff.

Participants represented several departments within the healthcare facilities. Their roles included patient care assistant, nurse, doctor, hospital administrator, lab tech, medical officer, pharmacist, and maintenance staff. Not all roles were represented in each FGD.

### Discussion guide

At baseline, the FGD topic guide included HH preferences, current HH practices in the context of the COVID-19 pandemic, HH products and infrastructure at the facilities, motivations and barriers to consistent HH practice, and perceived solutions to these barriers. These questions aimed to assess the day-to-day HH experiences of healthcare staff at the facilities and prompt discussion among FGD participants.

In the endpoint evaluation, the FGD guide covered similar topics of HH practices and preferences and included staff’s experiences with the intervention. Questions about the intervention explored the use and perceptions of the ABHR mounts and bottles that were provided throughout the healthcare facilities (improvements to the opportunity COM-B domain) and the training on HH that was administered electronically at all study facilities (improvements to the capability domain). The guide also included questions about suggestions or recommendations for the future, and how the COVID-19 pandemic has shaped HH awareness and practice (how the pandemic influenced the domain of motivation).

### Data collection

FGDs were conducted in English, the primary language of Belize, at the healthcare facilities at baseline and endpoint. For consistency, all discussions were moderated by the same male BCM research assistant who had received training in qualitative research and FGDs. To ensure anonymity, participants were assigned a number at the start of the discussion and referred to by their number (rather than their name) throughout the discussion. All discussions were audio-recorded and transcribed verbatim by the transcription service TranscribeMe (www.TranscribeMe.com, TranscribeMe Inc.). Each FGD lasted approximately 60 min.

### Coding and analysis

The codebooks for baseline and endpoint analyses were developed collaboratively by the FGD moderator and qualitative analysts from BCM and the U.S. Centers for Disease Control and Prevention (CDC). The coding structure was based on discussion questions, topic areas covered by the guide, and other themes and topics that arose in the transcripts. Five qualitative analysts completed the coding of all FGDs and conducted quality control by reviewing one another’s coding.

The data were coded using MAXQDA 2020 qualitative data analysis software (Verbi Software, Berlin, Germany 2020). A thematic analysis approach was used to identify key themes that emerged in the coded data.

### Intervention

**Figure 1 |:**
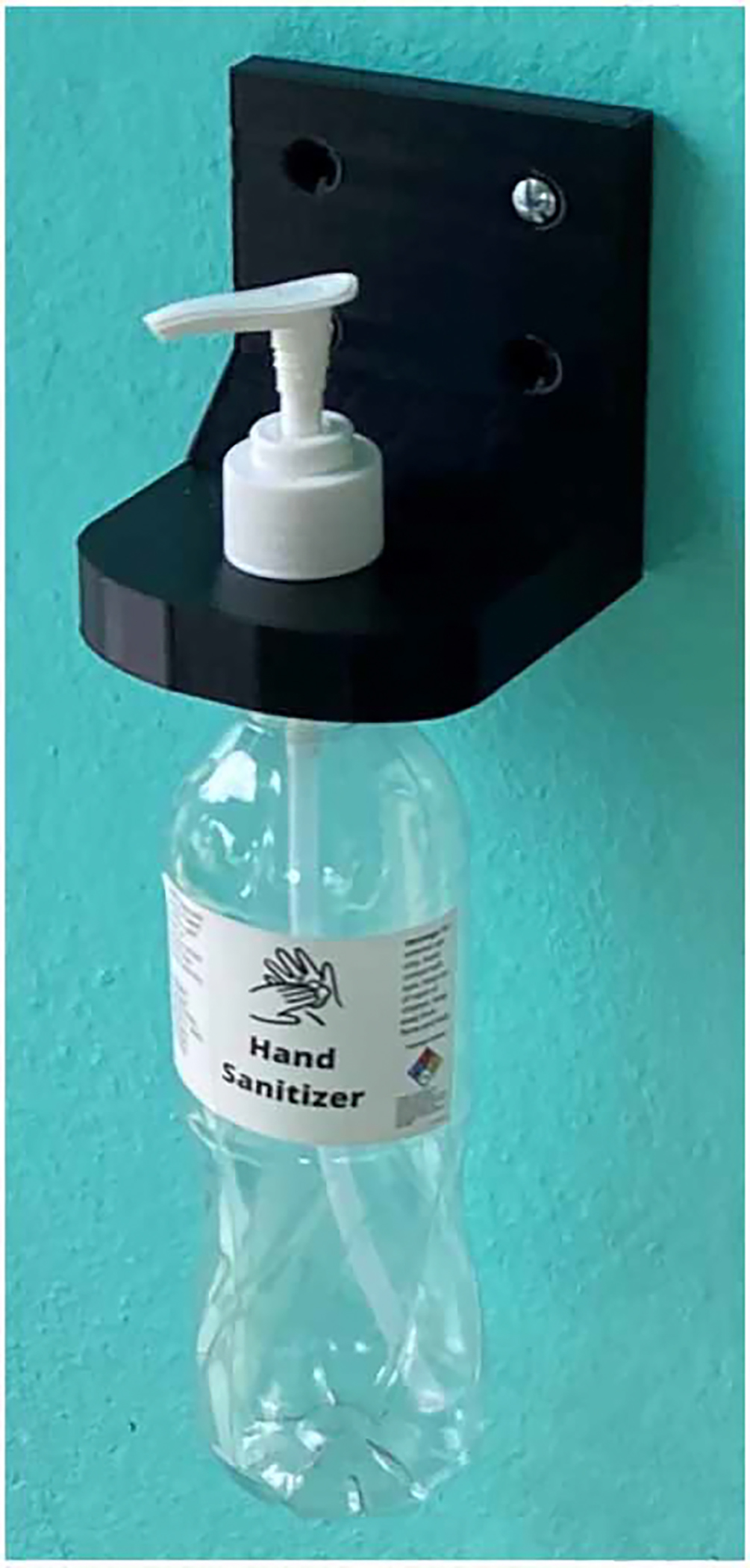
Wall-mounted ABHR bottle, with label provided to all facilities.

A two-part intervention, incorporating elements of the WHO multimodal HH improvement strategy, was launched at project sites in January–March 2022 (WHO 2009). To address ABHR access gaps, 3D-printed wall mounts and refillable ABHR bottles with labels were provided to each facility (Figure 1). Participating HCFs were instructed to fill the bottles with ABHR and install using the wall mounts in the vicinity of patient care areas where providers lacked consistent access to HH supplies. Facilities were responsible for providing their own ABHR, which may have differed between sites.

Additionally, an online training module on appropriate HH practices in healthcare settings was developed. The scenario-based training was designed by BCM and administered through the REDCap online platform (Harris et al. 2009). At project sites with limited internet connectivity, a PDF version of the training was distributed electronically or printed for staff to complete. Concepts from the World Health Organization’s *Five Key Moments of HH in Healthcare* were integrated into example scenarios with knowledge check questions and a final quiz to assess comprehension (WHO 2009). The completion of the training with a passing score of 70% was required by all clinical staff at the 11 facilities, and staff were able to retake the training and quiz as many times as needed to pass.

### Ethical considerations

The study was approved and determined to be non-research by the ethical review boards of BCM, CDC (Protocol ID: 0900f3eb81ca866a), and the Belize MOHW. Participation in FGDs was voluntary. Each participant provided verbal consent prior to starting the FGDs, and participants could decline involvement at any time. Audio recordings and deidentified transcripts were stored on a secured server and only accessed by members of the coding and analysis team.

## RESULTS

Themes that emerged at baseline and endpoint FGDs at healthcare facilities were feelings about HH, drivers and external cues to HH, barriers to HH, and HH education. Sentiments around these common themes changed from baseline FGDs to endpoint FGDs in some facilities and remained the same in others. Detailed findings from each of these themes are explored below. Three of the 11 sites did not install the ABHR mounts nor dispensers as intended, so those facilities did not provide feedback on that component of the intervention.

### Feelings about HH

At baseline, participants stated that having access to HH materials provided a feeling of safety, sense of security, and protection from the spread of illnesses such as COVID-19. A common sentiment among FGD participants was that being able to wash hands or use ABHR provided peace of mind that they were not infecting themselves or carrying viruses or infectious diseases to their household or to another area of the healthcare facility. Participants said that being able to use HH as a ‘method of prevention’ was comforting.

Both before and after the intervention, the preferences for one HH approach over the other varied between participants. At baseline, some participants preferred ABHR for its convenience. However, others reported that the alcohol content of the ABHR led to peeling and cracked skin on their hands and that they defaulted to handwashing with soap. Many expressed a preference for washing hands with soap and water, yet some shared that they disliked the harsh hand soap provided at their facilities.

‘But I rather wash my hand than sanitize [with] alcohol especially the one that they make here because it tends to dry my hands and irritate it.’ (Healthcare Facility 2, Baseline)

At endpoint preference for HH materials still varied, with some staff saying they preferred ABHR for its convenience and accessibility when rushed or on the go. However, other staff disliked ABHR due to the sticky residue that it left on their hands, the drying nature of the product, or because they felt the pump top ABHR bottles did not dispense enough ABHR. Some participants noted their belief that handwashing results in cleaner hands due to the residue ABHR leaves on the skin.

Participants expressed similar sentiments about access to HH products baseline and endpoint. They shared that they continued to use ABHR to prevent cross-contamination and to ensure a sterile environment for patients. Some participants said that they trusted the ABHR in their facility due to its strong odor of alcohol. During endpoint discussions, there was less concern mentioned about bringing home an illness to household members.

### Drivers and external cues to HH

At baseline, the most common motivator to practice HH among participants was protecting themselves, their patients, and their household members from illness. Additionally, participants commented that certain external cues served as reminders to practice proper HH to prevent the spread of COVID, including regular reminders over the hospital intercom and in the media, such as the radio or television. They repeatedly referenced signs, posters, and instructions at handwashing and ABHR stations throughout their healthcare facility that reminded staff of when and how to practice proper HH. Participants shared that these reminders made it impossible to not know how or when to practice HH. At endpoint, a principal motivation to practicing HH remained: to maintain the health of themselves, their household members, and the patients by not spreading or contracting disease.

‘Something we do have here at the hospital is [an] over the [intercom] system. They [do] their messages in every hour or hour and a half reminding basic measures including hand sanitizing, face mask usage, and physical distancing.’ (Healthcare Facility 4, Baseline)

‘But we have them [ABHR bottles] where necessary, now. We have more now.’ (Healthcare Facility 11, Endpoint)

‘It’s more convenient hand sanitizer, especially when there’s a lot of patients to see.’ (Healthcare Facility 1, Endpoint)

Participants also stated at baseline that when HH materials were more accessible, they were more likely to practice HH by washing or sanitizing their hands. Perceived convenience of HH stations varied greatly by facility and department in which personnel worked. In more than one facility, FGD participants reported providing their own personal ABHR, so that they had it available when needed. At endpoint, healthcare staff expressed in multiple FGDs that ABHR was more convenient for them between patient interactions and preferred it during times when their task load was heavy. They reiterated that convenience was an important driver of choosing to use ABHR. Regarding the new wall-mounted ABHR bottles, participants showed interest in the improved access to ABHR, the higher availability of ABHR throughout healthcare facilities, and the option to use different bottles within the installed mounts.

### Barriers to HH

FGD participants described multiple barriers that impact their ability to consistently practice HH. When asked about situations when they skip practicing HH despite knowing they should, limited time and lack of supplies consistently came up as the main obstacles, reflecting a gap in opportunity to practice.

At baseline, staff at some facilities shared that the available ABHR bottles in their facilities were sometimes broken or empty. A lack of supplies for HH was reported across multiple facilities from soap to paper towels. Some reported that when soap was available, it was often diluted past the point of usability. Multiple facilities reported having no designated personnel for the management of HH stations and supplies, so managers or supervisors would have to provide critical supplies from their own salaries and homes.

‘I know they dilute the soap that – especially, I notice it in the restroom. They dilute the soap.’ (Healthcare Facility 10, Baseline)

Some participants mentioned that although they preferred handwashing, there was often a lack of supplies needed to perform handwashing adequately, such as soap or paper towels for drying their hands. Some participants reported that in some areas, there were no wash basins for handwashing near patient consult rooms, so they had to use the closest bathroom’s handwashing station. However, if the bathroom was in use, they could not practice HH. Staff also reported that at times they did not feel able to fully wash or sanitize their hands due to lack of time or lack of access to an HH station. Some HCWs reported that they chose not to wash their hands between simple procedures because they did not feel it was necessary.

At endpoint FGDs, participants revealed that many infrastructure challenges related to handwashing stations remained the same as at baseline; they were not always located in the areas where HH was critical. After introducing the ABHR intervention, many participants mentioned that soap and hand sanitizer were still of insufficient supply and quality as well as reported finding ABHR bottles empty.

Some participants were able to identify a mechanism or point person to request HH supplies when they ran out. The point person for repairs varied by facility or participant’s personal process; these staff reportedly filled the role of maintenance, patient care assistant, infection control nurse, attendant, or maid. Other participants noted that there was no formal process for replacement, refill, or repair of dispensers.

### HH education

During the baseline FGDs, participants reported varied experiences with training and education on HH, with some reporting having received no formal training on it. One nurse shared that in nursing, school students were taught to use ABHR when it is more convenient, but to practice handwashing when hands are ‘physically soiled.’ A physician shared that they were taught about HH in medical school, but that they had not received additional training since then. Some participants stated that they took training with the infection control nurse at the beginning of the COVID-19 pandemic that included handwashing. One participant shared that the HCWs in their facility had taken refresher trainings on HH every 2 weeks since COVID-19 concerns arose, but others reported not receiving any previous HH training during the pandemic.

‘Kind of watching the advantages and disadvantages on which situation would you use the hand rubbing and the soap and water.’ (Healthcare Facility 1, Endpoint)

‘But [the training link] was giving [me] trouble – I tried to open it, but it wasn’t going. So I just left it like that.’ (Healthcare Facility 6, Endpoint)

Participants shared that they had enjoyed the interactive format of a past training MOHW provided, which included a product that stained their hands and showed where improved HH was needed on their own hands.

‘We have an infection control nurse, who is very active. She has a person that works under her, and that person is very active also, in making sure that the coworkers and the employees, they get that education. It’s not something that we started just yesterday. Ever since I started…10 years ago, well, we’ve been doing handwashing.’ (Healthcare Facility 8, Baseline)

‘When was the last time that you all’ve had HH training?’ – Moderator

‘Never.’ – Participant 1

‘I haven’t.’ – Participant 2

‘Med school.’ – Participant 3

(Healthcare Facility 3, Baseline)

In endpoint FGDs, after receiving a refresher training on HH as part of the intervention, participants stated that they felt more motivated and assured about their ability to have a continuum of care due to improved infection control in the health facility. Multiple participants stated they found the intervention training to be helpful and educational, especially the scenarios where they practiced identifying critical moments for HH. Most participants found the information to be elementary; it was perceived as a nice refresher by some, but as frustratingly mundane to others.

Some FGD participants shared that they were unable to complete the training for a variety of reasons: they were unaware it was offered, had technical issues opening or loading the virtual training, or felt it was too long and did not have time for it. Some Spanish-speakers noted having difficulty accessing the content as it was only offered in English.

### Staff suggestions and recommendations

As part of baseline FGDs, participants were asked for recommendations to improve HH in their facility at baseline before they were aware any intervention would be provided. Participants suggested interactive education in the form of practical training with spot checks to ensure staff were practicing HH. Spot checks took place after the training to monitor HCWs’ HH practice and provide feedback on timing, frequency, and if methods used were sufficient for infection prevention.

In critically reviewing the intervention during endpoint FGDs, participants shared they would find it helpful to receive continuing education credits for taking the intervention training. Many participants mentioned that incentives such as competitions within and between healthcare facilities would improve training uptake and HH practice in the future. Some staff offered that a champion badge would be a sufficient incentive to show pride in completion of the training. Staff also shared a desire for in-person trainings or practice scenarios rather than self-guided videos.

‘It would be nice if we could get credits off it because we need a certain amount of credits for the year of teaching.’ (Healthcare Facility 2, Endpoint)

## DISCUSSION

**Figure 2 |:**
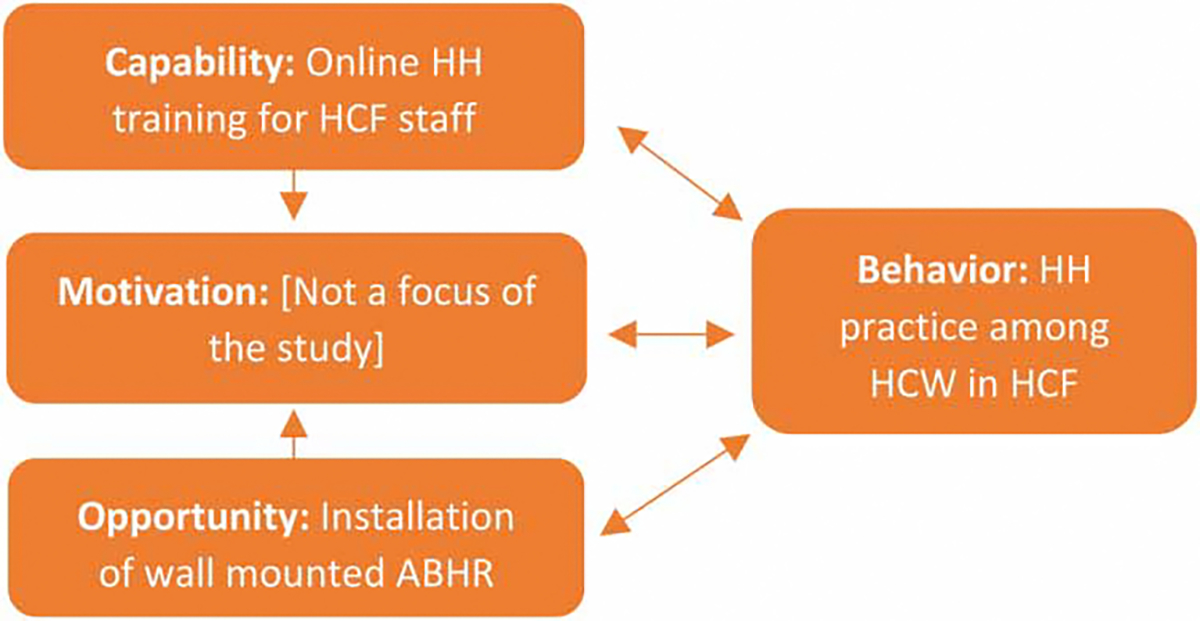
COM-B model.

These FGDs offer valuable insights into staff motivations and barriers to HH and experiences with a behavior change intervention designed to improve HH practice in the context of the COVID-19 pandemic (WHO 2009). Findings from the FGDs suggest that staff were motivated to practice HH by a desire to protect themselves, their patients, and their families. FGDs highlighted how challenges with HH product access and management, inconsistent quality of HH products, and gaps in knowledge were ongoing barriers, despite the implementation of a two-part HH intervention, which reflect continued short-comings within each of the behavioral driver domains of the COM-B model (see Figure 2). Implementation of both the wall-mounted ABHR bottles (improving opportunity to practice) and training (improving capability to practice) was inconsistent and may have limited the reach and impact of this intervention. The intervention did not focus on addressing motivation, which may have also limited the impact.

### Challenges with access and management

FGD participants at baseline and endpoint recognized convenience as an important factor in ABHR use. Among the facilities that received the full intervention, endpoint FGD participants noted the increased convenience of ABHR due to the installation of wall-mounted ABHR bottles. However, challenges persisted with maintaining a consistent supply of ABHR in the bottles, suggesting that the systems change approach of the WHO Multimodal HH Improvement Strategy was not fully actualized (WHO 2009).

These challenges show that increased accessibility of dispensers, on its own, is insufficient for improving use at points of care and that effective management systems are needed to ensure consistent access and facilitate HH practice. In some healthcare facilities, there were clear structures in place for requesting supply refills or repairs, but in many facilities the management structure for ABHR supplies was unclear or perceived to be nonexistent. Among those with a known structure, participants mentioned delays in getting requests filled, and there was little evidence of consistent supply monitoring at any sites.

A recent study described similar challenges in other LMIC contexts during the COVID-19 pandemic and recommended the implementation of stronger management systems to ensure access and thereby improve HH adherence (Berendes et al. 2022). To make these improvements, it is important to develop clear sets of standard operating procedures for managing and maintaining ABHR dispensers in the healthcare facilities and train designated staff on these processes. A study in Kenya also found that a lack of maintenance can lessen the impact of an HH intervention (Saito et al. 2017). Infection control nurses, maintenance staff, or custodial staff can support HH management, but it is important they have work time allocated for these tasks. In Uganda, a maintenance mechanism for ABHR management in healthcare facilities was successfully implemented, demonstrating the potential for such a system to help improve access to and management of ABHR in low-resource healthcare settings (Tusabe et al. 2023).

### Product quality issues

Beyond the issues of implementation and management, our findings suggest that product quality impacts the experience of HH practice among HCWs and may impact use. Staff described a sticky sensation on their hands when using hospital-produced ABHR, which caused them to dislike using it. This was also found in the Dominican Republic when hospitals produced ABHR onsite as part of a COVID-19 HH intervention (Schnorr et al. 2024). Similarly, some staff described concerns about the soap quality, describing the way it dried their hands; others felt ABHR dried their hands. It is important to address these concerns, along with concerns about diluted soap and lack of towels for drying hands, to ensure that staff feel confident using the HH products provided. Further research on staff perceptions of available HH products can inform recommendations to facilities on the best products to use.

### Knowledge gaps

Comments by participants in FGDs about not practicing HH after simple procedures suggest gaps in knowledge of WHO guidance for HH among HCWs in healthcare facilities. These recollections of their practices are reflected in HH observation data among staff at these same healthcare facilities during the COVID-19 pandemic, which showed that HH adherence in these facilities was 49% (Berendes et al. 2022).

Participant reports that they believed HH was not needed after minimal patient contact exposes a lack of understanding around the potential for contamination, pathways of exposure, and key moments for HH. Similar gaps in knowledge, such as the belief that HH was only needed after contact with bodily fluids, were also found in a review of literature in Latin America and the Caribbean (Delva et al. 2022). This also could be an indicator of HH fatigue as was found in the Dominican Republic and Guatemala when evaluating HH interventions during COVID-19 (Fahsen et al. 2024; Schnorr et al. 2024).

These knowledge gaps could be due, in part, to a lack of consistent HH education for HCWs in these facilities. Some FGD participants reported not having received education around HH since completing medical or nursing school and others shared the infection control nurse in their healthcare facility provided training at the start of the COVID-19 pandemic in 2020. Very few participants reported participating in a consistent training program within their facility. By applying HH training regularly within all facilities to all staff with patient interaction, staff will be better equipped to practice HH in the appropriate scenarios. Regular HH training would align with WHO’s recommended multimodal HH strategy, which incorporates continuing education and the improvement of HH in HCFs (WHO 2009).

### Inconsistent implementation

The potential impact of the intervention was limited in the three sites that did not install the wall mounts and ABHR bottles and affected the experiences of healthcare facility staff. Implementation science studies show that the delivery of an intervention as it was intended is crucial for the ‘successful translation of evidence-based interventions into practice’ (Breitenstein et al. 2010). Though the staff at the three sites without mounted ABHR bottles were offered the training component of the intervention, this alone may not lead to improved practice if supplies are still not available as intended. Consistent implementation is a key first step to management of wall-mounted ABHR.

### Low training uptake

Roughly half of the FGD participants reported not participating in the online or paper-based HH training, despite the MOHW making the training a requirement and sending monthly reminders. FGD participants stated a preference for in-person training in the future and spot checks of their HH practices. A study in Indonesia found that role model training, similar to the participatory style the HCWs in this study requested, for HH compliance among HCWs was more effective than the active presentation style of training or a combination of the two (Santosaningsih et al. 2017) and is recommended by WHO (2009). Spot checks could take place during and after the training to provide feedback on the effectiveness of HH methods used, timing HH practice, and the type of materials utilized.

Other training suggestions made by FGD participants included practice-based activities where participants use a UV fluorescent compound, such as Glo Germ, on hands prior to handwashing. After handwashing, UV light is used to identify parts of the hands that were not cleaned properly to demonstrate the need for rigorous handwashing and proper technique to remove contaminants from all surfaces. Studies have found this training approach to be effective for healthcare providers (Kısacık et al. 2021).

Healthcare staff also suggested providing incentives for those who partake in any training that is made available to them. They suggested various examples of what these incentives have been in the past and what they could be for future training opportunities; examples included low-cost options such as ‘Champion’ badges or completion stickers. A study in Dutch nursing homes found that HCWs increased their HH adherence when nonfinancial incentives were part of a multimodal intervention approach (Teesing et al. 2020).

To improve HH practices and reduce infections, educational interventions must be tailored to the cultural context (Delva et al. 2022). Delva et al. found that community research designed around the existing culture is needed, as well as WASH-focused government regulations to ensure HH supplies are sufficient (Delva et al. 2022). Any approach that would garner greater participation in training would help reach the WHO’s Multimodal HH Improvement Strategy’s education component (WHO 2009).

### Limitations

There are several limitations to this study. FGDs were formed using a convenience sample where the role of participants in the facility was not a criterion for inclusion; participants and groups may not have been representative of the workforce. FGD participants may have been hesitant to voice concerns in front of their professional superiors, especially as social hierarchy is an important cultural factor within much of Central America and the Caribbean. The moderator of all FGDs was a BCM staff member and FGD participants knew BCM was responsible for the intervention. This may have biased the responses of those who participated in the FGDs.

Additionally, the endpoint FGDs were conducted with the assumption that all 11 participating healthcare facilities had implemented the intervention as designed. However, as mentioned, in 3 of the 11 facilities, ABHR wall mounts and bottles were not installed, so these participants’ experiences did not include the same increased access to ABHR. Online delivery of the education component of the intervention was not ideal for this setting, especially in areas with limited internet access. Participants’ suggestions around how to improve the training may have been impacted by the lack of participation. Additionally, incorporating other components from the WHO’s Multimodal HH Improvement Strategy could improve HCWs’ experiences with HH interventions (WHO 2009). Due to the limited study timeframe, there was no opportunity to expand activities to include the WHO HH Improvement Strategy in future action plans. Furthermore, as the COVID-19 pandemic continued, fears of contracting illness or routines to prevent illness may have waned. Finally, these FGDs may not reflect the experiences or beliefs of HCWs in private, smaller, and rural public healthcare facilities.

## CONCLUSION

Improving access to HH resources and increasing knowledge of proper HH practices persisted as challenges in healthcare facilities in Belize following an intervention aimed at increasing access to ABHR and training HCWs on correct HH practices. Further research on how to improve HH practices among HCWs in Belize and improve access to the supplies needed for those methods may have a positive impact on HH practices. A practical starting point would be to incorporate the other components of the WHO’s HH Improvement Strategy that were not included in this study. Findings from this qualitative component, in conjunction with additional findings from the larger evaluation which included HH observations and increased access to HH supplies, will provide a more comprehensive evaluation of this work and inform future HH interventions in the Belizean public healthcare context. Future work should incorporate these qualitative findings with other findings from the broader study.

## Data Availability

Data cannot be made publicly available; readers should contact the corresponding author for details.;.

## HIGHLIGHTS

- Self-reported barriers to hand hygiene practice among healthcare workers at 11 facilities in Belize included insufficient time, inconsistent access to materials, and gaps in education.
- An intervention was designed and implemented with two aims: to increase access to alcohol-based hand rub and to provide online hand hygiene training for healthcare workers in the 11 sites.
- Healthcare staff experiences with the intervention were mixed, highlighting the importance of tailoring interventions to context and learning preferences.
